# Essential roles of buried phenylalanine in the structural stability of thioredoxin from a psychrophilic Arctic bacterium *Sphingomonas* sp.

**DOI:** 10.1371/journal.pone.0261123

**Published:** 2021-12-15

**Authors:** Thu-Thuy Nguyen, Trang Hoang, Kiet N. Tran, Hyeonji Kim, Sei-Heon Jang, ChangWoo Lee

**Affiliations:** Department of Biomedical Science and Center for Bio-Nanomaterials, Daegu University, Gyeongsan, South Korea; Consiglio Nazionale delle Ricerche, ITALY

## Abstract

Thioredoxin (Trx), a small redox protein, exhibits thermal stability at high temperatures regardless of its origin, including psychrophiles. Trxs have a common structure consisting of the central β-sheet flanked by an aliphatic cluster on one side and an aromatic cluster on the other side. Although the roles of aromatic amino acids in the folding and stability of proteins have been studied extensively, the contributions of aromatic residues to the stability and function of Trx, particularly Trxs from cold-adapted organisms, have not been fully elucidated. This study examined the roles of aromatic amino acids in the aromatic cluster of a Trx from the psychrophilic Arctic bacterium *Sphingomonas* sp. PAMC 26621 (SpTrx). The aromatic cluster of SpTrx was comprised of W11, F26, F69, and F80, in which F26 at the β2 terminus was buried inside. The substitution of tyrosine for F26 changed the SpTrx conformation substantially compared to that of F69 and F80. Further biochemical and spectroscopic investigations on F26 showed that the F26Y, F26W, and F26A mutants resulted in structural instability of SpTrx in both urea- and temperature-induced unfolding and lower insulin reduction activities. The Trx reductase (SpTR) showed lower catalytic efficiencies against F26 mutants compared to the wild-type SpTrx. These results suggest that buried F26 is essential for maintaining the active-site conformation of SpTrx as an oxidoreductase and its structural stability for interactions with SpTR at colder temperatures.

## Introduction

Thioredoxin (Trx) is a class of small redox proteins (~12 kDa) that protect cells against oxidative stress [1, 2]. Oxidized thioredoxin (Trx-S_2_) is reduced to Trx-(SH)_2_ by thioredoxin reductase (TR) using reduced nicotinamide adenine dinucleotide phosphate (NADPH) as an electron donor [3, 4]. The reduced Trx passes electrons to many cytoplasmic proteins, including ribonucleotide reductase [5] and methionine sulfoxide reductase [6]. Trx consists of five antiparallel β-strands sandwiched by a pair of two α-helices, which include the canonical Trx fold—four β-strands surrounded by three α-helices [2]. The Trx fold is common to enzymes that catalyze the dithiol-disulfide exchange and is highly conserved from archaea to mammals [7, 8]. Trx contains a redox-active CXXC catalytic motif [7]. The sequence reconstruction of ancestral thioredoxins (Trxs), which goes back approximately four billion years, shows a robust structure and high thermal stability [9, 10].

The structures of modern Trxs are less stable than ancestral Trxs [9, 10]. Modern *Escherichia coli* Trx (EcTrx) has a longer loop between α1-helix and β2-strand than with thermophilic Trxs [11]. EcTrx also displays enhanced flexibility of the α3-helix at the expense of reduced flexibility of the α4-helix while core β-sheet rigidity is maintained [12]. Trxs from psychrophilic bacteria exhibit both a lower melting temperature (*T*_m_) and lower urea concentrations for unfolding compared to Trxs from thermophilic and mesophilic bacteria [10]. On the other hand, all known Trxs show overall high thermal stability, including psychrophilic *Pseudoalteromonas haloplanktis* (PhTrx), which has a half-inactivation time of 4.4 h at 95°C [13]. In contrast to Trxs, TRs from psychrophilic bacteria show the typical characteristics of cold-adapted enzymes with heat-labile structures [13, 14].

**Fig 1:**
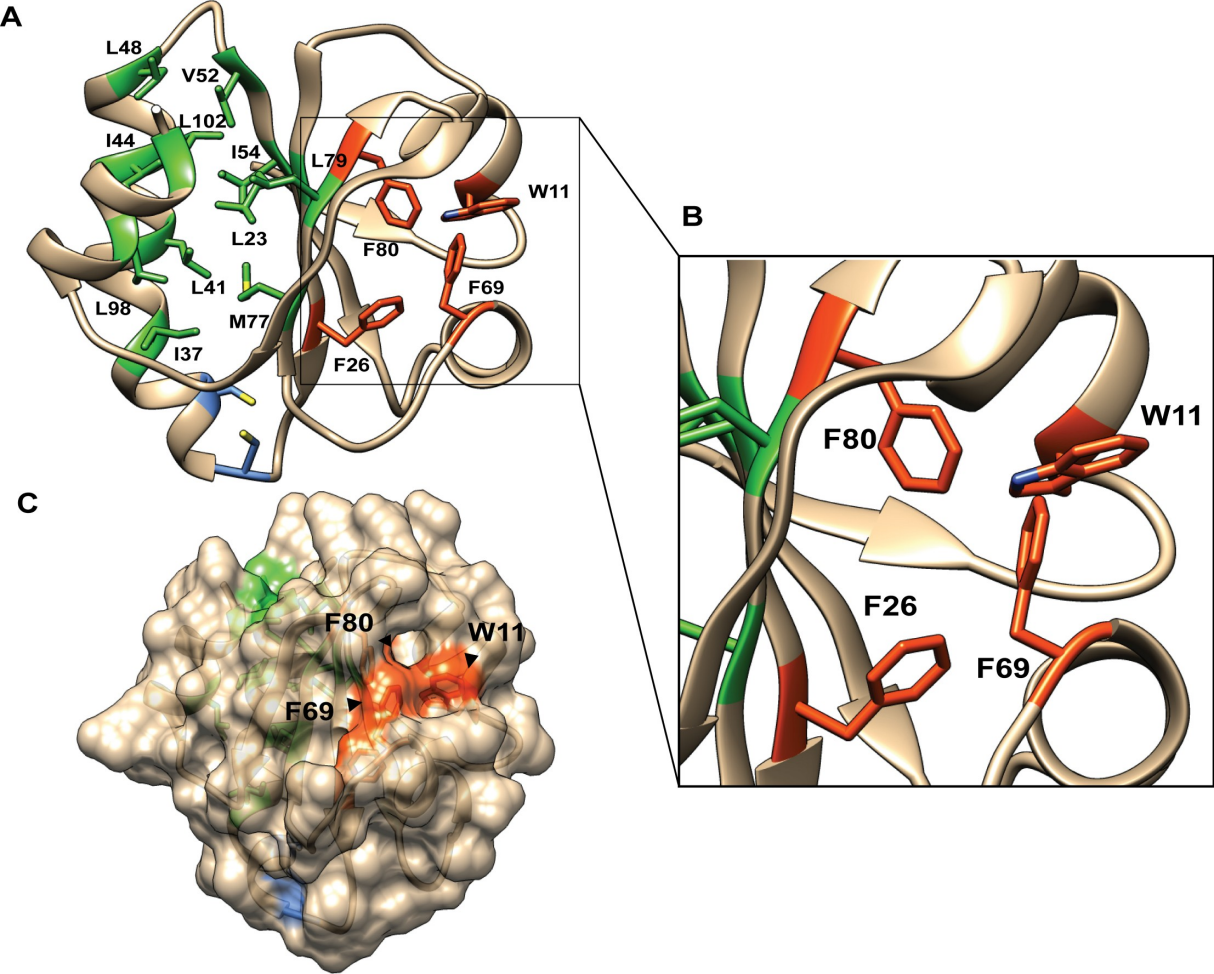
Structural model of SpTrx. (A) The hydrophobic core of SpTrx. The central β-sheet of SpTrx divides its hydrophobic core into two clusters: one comprising aliphatic residues (green) and the other comprising aromatic residues (orange). Two catalytic cysteine residues are shown in blue. (B) Enlarged view of the SpTrx aromatic cluster. W11, F26, F69, and F80 are positioned in a tetrahedral geometry. (C) Surface view of SpTrx. W11, F69, and F80 are located on the surface of SpTrx, whereas F26 is buried inside.

The Trx scaffold delimits its hydrophobic core into two clusters on either side of the central β-sheet, with one comprising aliphatic residues (“aliphatic cluster”) and the other comprising aromatic residues (“aromatic cluster”) [7, 15] (Fig 1A). The L42 and L78 residues in the aliphatic cluster of EcTrx are in contact, and non-conservative mutations of L42 and L78, including the L78 to lysine or arginine mutation, have little effect on the function of EcTrx [16, 17]. On the other hand, a mutation of F25 in the aromatic cluster of *Rhodobacter sphaeroides* Trx (RsTrx) to aliphatic side chains (valine, leucine, and isoleucine) increases the susceptibility to urea-induced unfolding, whereas a mutation of F25 to tyrosine leads to a slightly less stable structure compared to the wild-type (WT). However, those studies reported mesophilic Trxs, which means the roles of hydrophobic amino acids in the cold adaptation of Trxs have not been fully studied.

This study examined how the selection of aromatic amino acid residues affects the stability and function of Trx from the psychrophilic Arctic bacterium *Sphingomonas* sp. PAMC 26621 (SpTrx). The aromatic cluster of SpTrx comprised a tetrahedral geometry of one tryptophan and three phenylalanine residues (W11, F26, F69, and F80), in which F26 at the β2 terminus is buried inside (Fig 1B). In the present study, SpTrx WT exhibited a heat-stable structure despite originating from a psychrophilic Arctic bacterium, whereas its Trx reductase (SpTR) showed typical properties of the cold-adapted enzyme. Since F26 was highly conserved in Trxs from diverse working temperatures and positioned in a hydrophobic environment, this study examined the effects of F26 mutations, particularly at the aromatic residues (tyrosine and tryptophan) and the smallest hydrophobic residue (alanine), on the cold adaptation of SpTrx, its interaction with SpTR, and oxidoreductase activity.

## Materials and methods

### Materials

*Sphingomonas* sp. PAMC 26621 was provided by the Polar and Alpine Microbial Collection of the Korea Polar Research Institute (Incheon, South Korea) [18]. The pET28 expression vector was purchased from Novagen (Madison, WI, USA). The TA vector, restriction enzymes, and *pfu* polymerase were acquired from Enzynomics (Daejeon, South Korea). HisTrap, Capto Q, HiPrep desalting 26/10, Superdex 200 10/300 GL, and Superdex 200 prep grade XK16 columns were purchased from GE Healthcare (Piscataway, NJ, USA). 4,4′-Dianilino-1,1′-binaphthyl-5,5′-disulfonic acid (bis-ANS) was purchased from Sigma (St. Louis, MO, USA). All other chemical reagents were purchased from Sigma or Tokyo Chemical Industry (Tokyo, Japan).

### Bioinformatics

The structural model of SpTrx (NCBI ID: WP_010164143.1) was constructed at the Swiss-Model based on the crystal structure of *Acetobacter aceti* Trx (PDB ID: 2I4A) [19]. Multiple sequence alignment of SpTrx with Trxs from psychrophilic, mesophilic, and thermophilic bacteria was performed using Clustal Omega [20].

### Gene cloning and site-directed mutagenesis

The 324-bp *trxA* (*sptrx*) and 966-bp *trxRB* (*sptr*) genes were amplified from the genome of *Sphingomonas* sp. PAMC 26621 by polymerase chain reaction (PCR) and subcloned into a TA vector, respectively. The TA-*sptrx* construct, digested by *Nco* I and *Hind* III, and the TA-*sptr* construct, digested by *Nco* I and *Xho* I, were subcloned into a pET28 vector, respectively. The pET28-*sptrx* construct was used as a template for site-directed mutagenesis by PCR using *pfu* polymerase (PCR primers in S1 Table). Three phenylalanine residues (F26, F69, and F80) in the SpTrx aromatic cluster were replaced with tyrosine (F26Y, F69Y, and F80Y). Mutations of F26 to tryptophan and alanine generated two additional mutants (F26W and F26A). PCR products were incubated with *Dpn* I at 37°C for 1 h before transformation into *E*. *coli* BL21 (DE3). The accuracy of mutant plasmids was confirmed by DNA sequencing.

### Expression and purification of SpTrx WT and mutant proteins

A single colony of *E*. *coli* BL21 (DE3) was grown overnight in a Luria-Bertani (LB) medium with 100 μg/mL of kanamycin. The overnight culture was inoculated into 200 mL of freshly prepared LB medium (1:100 dilution) containing kanamycin. After reaching an optical density of 0.6–0.8 at 37°C, isopropyl β-D-1-thiogalactopyranoside (1 mM final concentration) was added to the LB medium, and the culture was grown for an additional 4 h at 30°C. The cells were harvested by centrifugation and washed with buffer A (50 mM Tris·HCl, 50 mM NaCl, 5 mM imidazole, and 0.1 mM ethylenediaminetetraacetate [EDTA]). Cell pellets containing SpTrxs or SpTR were resuspended in buffer A, followed by sonication on ice. The supernatant after centrifugation was loaded into a 1-mL HisTrap column equilibrated with buffer A. The non-specific proteins were washed with buffer A containing 20 mM imidazole, followed by elution of the target protein by buffer A containing 300 mM imidazole. All fractions containing SpTrxs or SpTR were pooled and loaded into a 5-mL Capto Q column on an AKTA explorer system (GE Healthcare). The recombinant Trx proteins were eluted with a linear gradient of 50–500 mM NaCl in buffer B (50 mM Tris·HCl and 50 mM NaCl). The recombinant TR protein was desalted in buffer C (50 mM potassium phosphate, pH 8.0, and 50 mM NaCl) by HiPrep 26/10 column chromatography, followed by elution through a Superdex 200 prep grade XK16 column. All fractions containing the target proteins were pooled. The purified SpTrxs and SpTR proteins were kept in buffers B and C, respectively, and stored at –80°C until needed.

The molecular weights of SpTrxs and SpTR were analyzed by gel filtration chromatography using a Superdex 200 10/300 GL column in buffer D (50 mM sodium phosphate, pH 7.2, and 100 mM NaCl). A protein standard mix (15–600 kDa) from Sigma was used to calculate the molecular weights of SpTrx and SpTR (bovine thyroglobulin 670 kDa, γ-globulin 150 kDa, albumin 44.3 kDa, and ribonuclease A 13.7 kDa).

### Fluorescence spectroscopy

Intrinsic protein fluorescence measurements were performed to evaluate the temperature-induced protein unfolding, urea-induced unfolding, and acrylamide-mediated quenching of protein fluorescence using a Scinco FS-2 fluorescence spectrometer (Seoul, South Korea) (excitation at 280 nm and emission between 300–400 nm). Because the tryptophan fluorescence of SpTrx (excitation at 295 nm) was low, both tyrosine and tryptophan were excited at 280 nm, and the fluorescence intensity was normalized to 100%. Purified SpTrx existed mainly in an oxidized form, and the addition of dithiothreitol increased the fluorescence of SpTrx (S3 Fig), as shown previously for EcTrx [21]. The temperature-induced protein unfolding of WT SpTrx and SpTR was evaluated by measuring the protein fluorescence at 25°C after incubating the proteins at various temperatures for 2 h in buffer B. Urea-induced protein unfolding was measured for 10 μM SpTrx WT and F26 mutant proteins after incubation with different concentrations of urea (0–10 M) in buffer E (100 mM potassium phosphate, pH 7.0, and 150 mM NaCl) at 25°C for 30 min.

For the acrylamide-induced quenching of protein fluorescence measurements, proteins were incubated at various temperatures (4, 30, and 50°C) for 2 h in buffer B. After incubation, acrylamide (0–0.5 M) was added to buffer B for 2 min at 25°C, and the fluorescence was measured. Acrylamide Stern-Volmer plots are presented as the ratio of intrinsic fluorescence intensity in the absence of acrylamide (F_0_) to fluorescence intensity in the presence of 0.1–0.5 M acrylamide (F). The Stern-Volmer plot illustrated a trend for the slight upward curves as plots of F_0_/F versus [Q]. Thus, the modified Stern-Volmer equation (F_0_/F = 1+ *K*_D_[Q]) was used in these cases, where Q is the acrylamide concentration, and *K*_D_ is the Stern-Volmer constant calculated from the slope of the linear region of the quenching curve.

The temperature-induced unfolding of SpTrx WT and F26 mutants was evaluated by measuring the bis-ANS fluorescence. Ten μM proteins in buffer C were incubated at various temperatures (4, 30, 50, 70, and 90°C) for 2 h before being added to the reaction mixture containing 10 μM bis-ANS in buffer C. After 30 min of incubation, the fluorescence spectra of the samples were measured at 25°C using a Scinco FS-2 fluorescence spectrometer (excitation at 385 nm and emission at 400–600 nm).

### Protein thermal shift analysis

A thermal shift assay using SYPRO orange dye was performed on an Applied Biosystems StepOnePlus real-time PCR instrument in the 25–99°C range in 1°C/min increments. The proteins (0.6 mg/mL) were added to a phosphate buffer (100 mM sodium phosphate, pH 7.2 and 100 mM NaCl) containing 10× SYPRO orange dye in a total volume of 20 μL. The *T*_m_ value, at which 50% of the protein was unfolded, was determined using Protein Thermal Shift software v1.4 (Applied Biosystems).

### Circular dichroism (CD) spectroscopy

The samples at a final concentration of 0.3 mg/mL in buffer B were incubated at different temperatures (4, 50, and 90°C) for 2 h. The CD spectra were measured using a JASCO J-1500 spectropolarimeter at the Korea Basic Science Institute (Ochang, Korea). The results show residual ellipticity (mdeg) versus wavelength (nm) using GraphPad Prism software. The αα-helical content of each protein was calculated using the K2D3 server [22].

### Trx reductase assay and kinetics

The assay mixture (50 mM potassium phosphate, pH 8.0, 50 mM NaCl, 5 mM 5,5-dithio-bis-2-nitrobenzoic acid (DTNB), and 180 mM NAPDH) was incubated with 50 nM SpTR and a range of Trxs (0.1–10 μM) at 4 or 30°C for 3 min. The SpTR activity was measured from the reduction of DTNB at 412 nm. One enzyme unit is defined as the amount of enzyme catalyzing the formation of 1.0 μM of 1,3,5-trinitrobenzene per min. The Michaelis–Menten constant (*K*_m_) and the catalytic rate constant (*k*_cat_) were calculated by using the double reciprocal Lineweaver–Burk plot.

### Insulin reduction assay using SpTrx WT and mutants

Insulin (0.13 mM) and an appropriate amount of SpTrx proteins were added to the reaction mixture (100 mM sodium phosphate, pH 7.0, 2 mM EDTA, and 0. 33 mM dithiothreitol) to a final volume of 800 μL. A negative control was set up without the Trx protein. The mixtures were incubated at 25°C and measured at 10 min intervals for up to 100 min by recording the absorbance of the precipitated β-chain of insulin at 650 nm using a Shimadzu UV-1800 spectrophotometer (Kyoto, Japan).

## Results

### Structural model of SpTrx and sequence analysis

The structural model of SpTrx based on the crystal structure of mesophilic *Acetobacter aceti* Trx (54.2% amino acid sequence identity) showed a typical Trx fold with five β-strands sandwiched on both sides by a pair of α-helices (Fig 1A). The catalytic redox motif of SpTrx consisted of the characteristic WCGPC sequence [1]. The hydrophobic core of SpTrx displayed two clusters, one consisting mainly of aliphatic amino acids (L23, I37, L41, I44, L48, V52, I54, M77, L79, L98 and L102) (Fig 1A) and the other comprising a tetrahedral configuration of four aromatic amino acids (W11, F26, F69, and F80) (Fig 1B). The four aromatic residues were highly conserved in Trxs adapted to a wide range of temperatures (S2 Fig). The first aromatic residue, W11, with the indole ring located perpendicular to the protein surface, did not form a hydrogen bond via the indole N–H moiety (Fig 1B). The second aromatic residue, F26, was buried inside, and the other residues (F69 and F80) were located near the protein surface (Fig 1B and 1C).

### Protein expression and purification of SpTrx and SpTR

**Fig 2:**
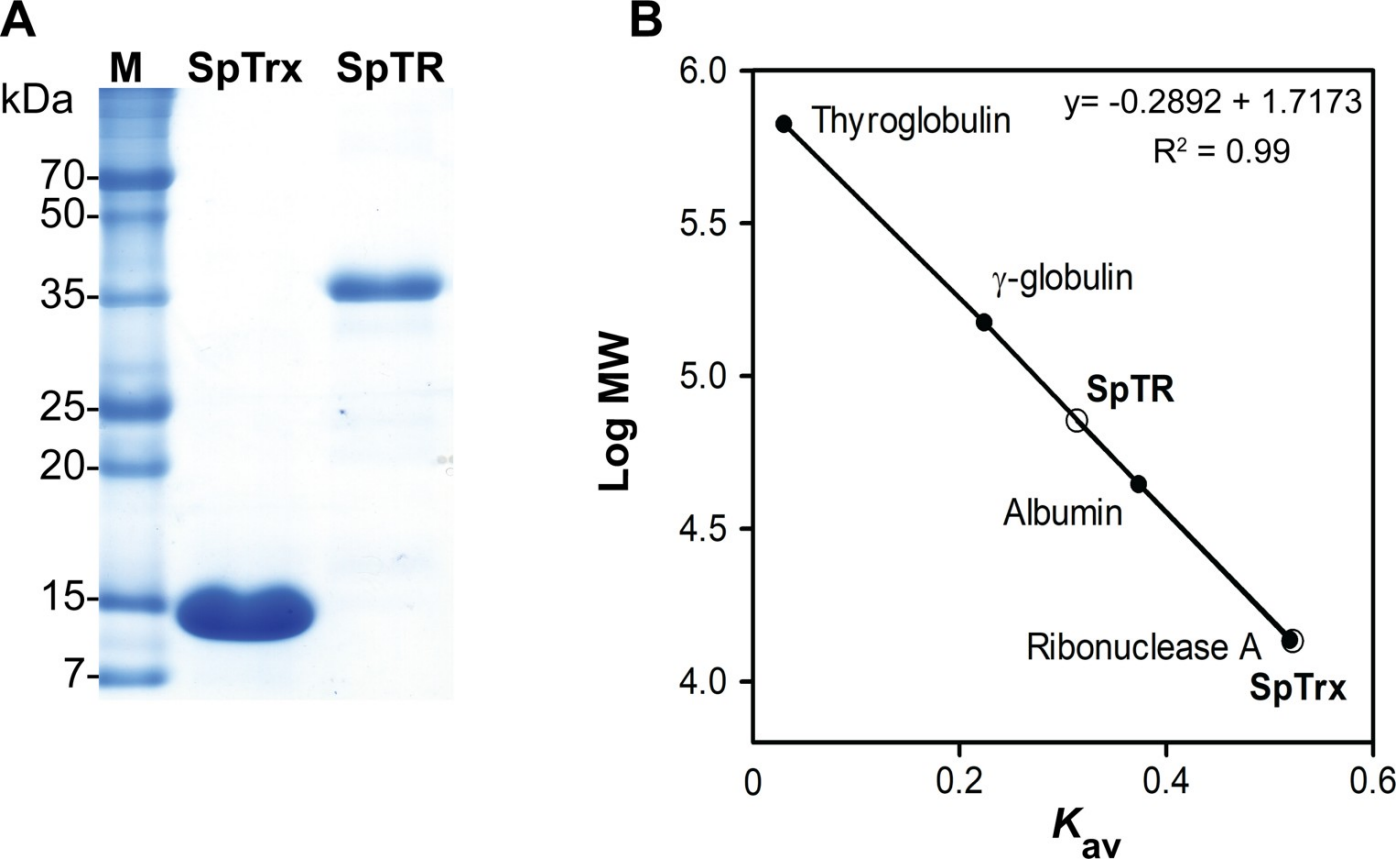
Purification and molecular weight determination of WT SpTrx and SpTR. (A) SDS-polyacrylamide gel electrophoresis of WT SpTrx and SpTR after protein purification. (B) Determination of the molecular weight of native SpTrx and SpTR by size-exclusion chromatography on a Superdex 200 10/300 GL column. Thyroglobulin (667 kDa), γ-globulin (150 kDa), albumin (44.3 kDa), and ribonuclease A (13.7 kDa).

The recombinant SpTrx (107 amino acids) and SpTR (321 amino acids) proteins with a C-terminal six histidine-tag are expressed in *E*. *coli* BL21 (DE3) as soluble proteins. The proteins were purified by nickel-chelate affinity chromatography, followed by Capto Q anion-exchange chromatography. SpTR was purified further to homogeneity by Superdex 200 gel filtration chromatography (Fig 2A). SpTrx appeared as a monomer (13.7 kDa) in solution whereas SpTR appeared as a dimer (71.6 kDa), as determined by Superdex 200 gel filtration chromatography (Fig 2B).

### Thermal stability and conformational flexibility of WT SpTrx and SpTR

**Fig 3:**
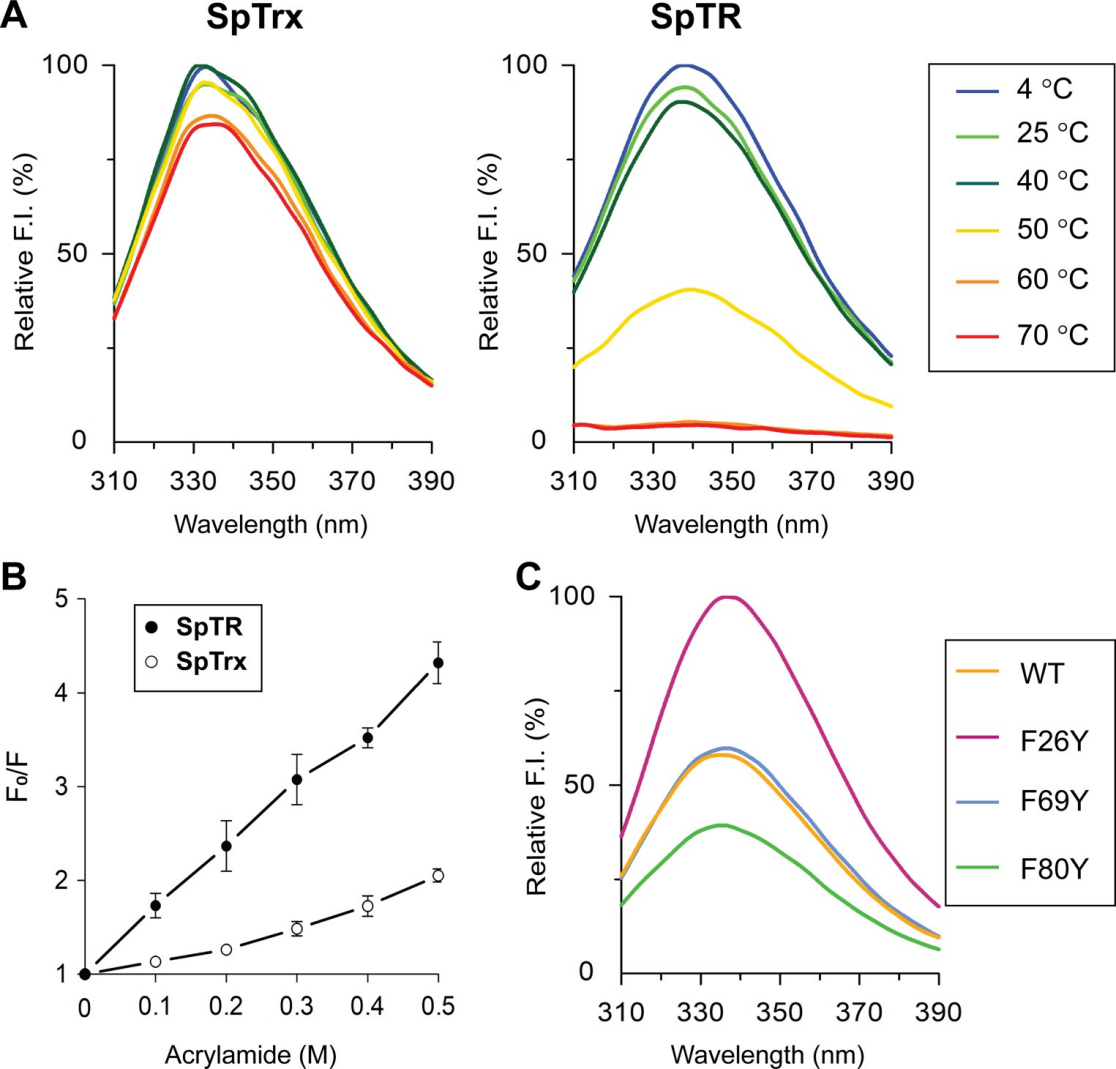
Comparison of the thermal stability and conformational flexibility of two components of Trx system in *Sphingomona*s sp. PAMC 26621 and the fluorescence properties of phenylalanine mutants of SpTrx. (A) Fluorescence spectra of WT SpTrx and SpTR upon incubation of the proteins at various temperatures (4–70°C) for 2 h. (B) Acrylamide Stern-Volmer plot for WT SpTrx and SpTR. F_0_, the maximum fluorescence intensity without acrylamide; F, the maximum fluorescence intensity under increasing concentrations (0.1–0.5 M) of acrylamide. (C) Effect of tyrosine substitution in the aromatic cluster of SpTrx on protein fluorescence. The data are presented as the means of three experiments (A and C) or means ± S.D. of three experiments (B). Excitation at 280 nm.

The temperature-induced unfolding of SpTrx and SpTR was evaluated by measuring the intrinsic protein fluorescence (excitation at 280 nm) upon incubation of proteins at 4–70°C for 2 h, respectively. SpTrx has four tryptophan and no tyrosine residues, whereas SpTR has four tryptophan and eight tyrosine residues. Although SpTrx maintained its tertiary structure within the 4–70°C range, SpTR began to lose its tertiary structure from 50°C onwards and lost its structure at 60°C (Fig 3A). The optimal temperature of SpTR was 30°C (S4 Fig).

The conformational flexibilities of SpTrx and SpTR were compared using the acrylamide-induced quenching of protein fluorescence. The acrylamide Stern-Volmer plot indicated greater flexibility for SpTR than SpTrx, as shown in the F_0_/F value (Fig 3B). The *T*_m_ values measured by SYPRO orange-based protein thermal shift analysis showed that SpTrx has a *T*_m_ value of 73.4°C while SpTR has a *T*_m_ value of 54.0°C (Table 1). Overall, SpTrx maintained a heat-stable structure despite its psychrophilic origin, whereas SpTR exhibited a heat-labile flexible structure similar to the other cold-adapted enzymes.

**Table 1:** *T*_m_ values of SpTrx and SpTR proteins.

	*T*_m_ (°C)
SpTrx WT	73.4 ± 0.2
F26Y	63.5 ± 0.9
F26W	59.3 ± 1.0
F26A	56.4 ± 1.0
F69Y	74.0 ± 0.1
F80Y	67.0 ± 0.1
SpTR WT	54.0 ± 1.0

*T*_m_ values were determined by SYPRO orange-based thermal shift analysis using real-time PCR. The data presented are the means ± S.D. of three experiments.

### Effect of tyrosine substitutions in the aromatic cluster of SpTrx on protein fluorescence

Phenylalanine generally contributes little to the intrinsic fluorescence of proteins because of its low absorption and quantum yield [23]. The role of phenylalanine residues in the aromatic cluster of SpTrx was examined by fluorescence spectroscopy using similarly sized tyrosine with its hydroxyl group substituted for each of the three phenylalanine residues (F26, F69, and F80). Interestingly, among the three mutants, the fluorescence intensity of F26Y was the highest, as evidenced by a 1.7-fold increase compared to that of WT (Fig 3C). F69Y had an insignificant effect on protein fluorescence, whereas F80Y showed reduced fluorescence, with a 0.67-fold intensity compared to WT. The increased fluorescence intensity of F26Y despite the hydrophilic characteristics of the tyrosine hydroxyl group indicated that F26 was positioned in a hydrophobic environment. F26Y and F80Y showed 6–10°C lower *T*_m_ values (63.5°C and 67.0°C, respectively), suggesting that the presence of a tyrosine hydroxyl group at positions 26 and 80 caused structural instability (Table 1). F69Y had little effect on its *T*_m_ value.

### Urea-induced unfolding of SpTrx WT and the mutants

**Fig 4:**
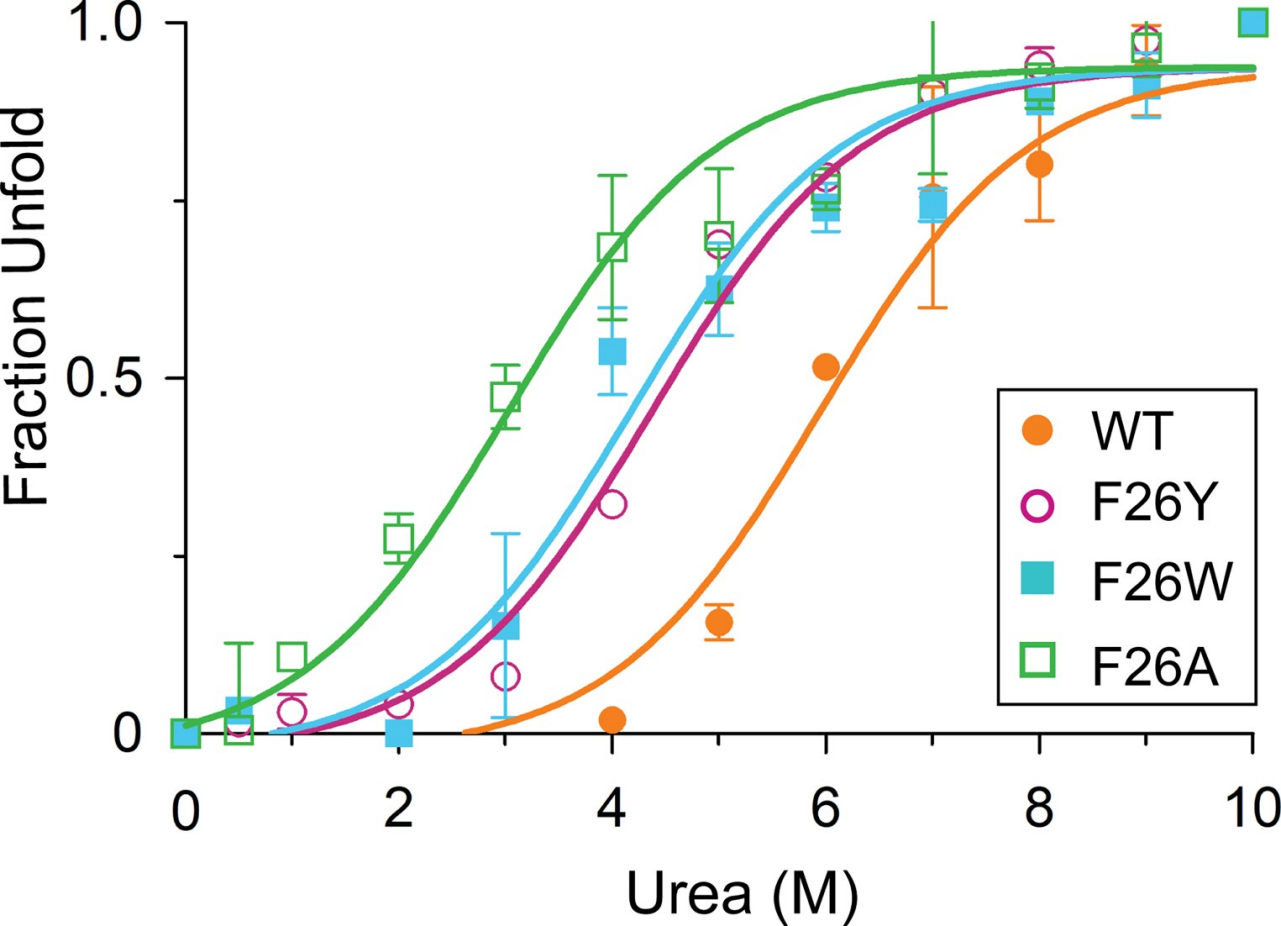
Urea-induced unfolding curves of SpTrx WT and F26 mutants. Fluorescence spectra were measured after incubating the proteins with different concentrations of urea (0–10 M) at 25°C for 30 min (excitation at 280 nm). Data presented are means ± S.D. of three experiments.

The roles of buried phenylalanine (F26) on the structural stability of SpTrx were investigated further by generating two more F26 mutants (F26W and F26A) by site-directed mutagenesis (S1 Fig). Although F26W was expressed as a soluble protein, F26A was expressed mostly as inclusion bodies, and only a small fraction was obtained as soluble proteins (data not shown). Urea-induced unfolding was performed to evaluate the structural stabilities of the SpTrx WT and F26 mutants with increasing urea concentration (0–10 M) at 25°C. SpTrx WT was the most stable, while F26A was susceptible to denaturation at low urea concentrations (Fig 4). F26Y and F26W showed similar stabilities from the urea-induced unfolding curves (Fig 4). The results suggest that the presence of a tyrosine hydroxyl group or indole N–H moiety in the aromatic cluster of SpTrx led to reduced chemical stability compared to purely hydrophobic phenylalanine.

### Thermal stabilities of SpTrx WT and the mutants using bis-ANS fluorescence

**Fig 5:**
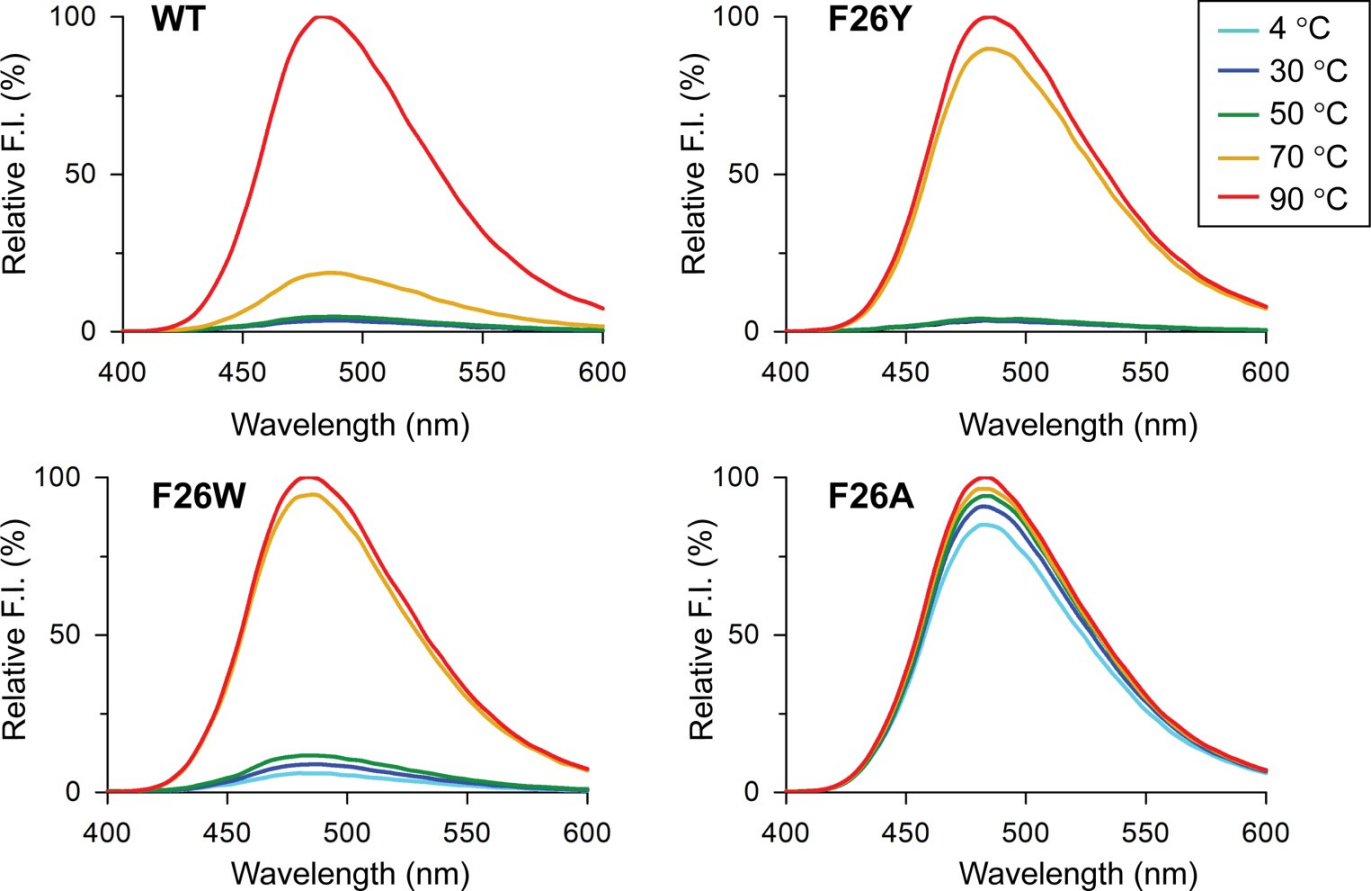
Bis-ANS fluorescence of SpTrx WT and F26 mutants. Temperature-induced unfolding of SpTrx WT and F26 mutants were measured using bis-ANS fluorescence after incubating the proteins (10 μM) at various temperatures (4–90°C) for 2 h (excitation at 385 nm). The data presented are the means of three experiments.

The thermal stabilities of the SpTrx WT and F26 mutants were compared using bis-ANS, which binds to the hydrophobic region of proteins, after incubating the proteins at various temperatures (4–90°C) for 2 h. WT, F26Y, and F26W maintained their overall tertiary structures in the 4–50°C temperature range (Fig 5). On the other hand, the fluorescence intensities of F26Y and F26W were elevated substantially at 70°C compared to that of WT. In contrast, F26A showed higher bis-ANS fluorescence at all temperatures, including at 4°C. The SpTrx WT and F26 mutant proteins were denatured at 90°C. The results suggest that the stability of the SpTrx WT and F26 mutants was in the order of WT > F26Y > F26W > F26A. Protein thermal shift analysis confirmed approximately 10–17°C lower *T*_m_ values for the F26 mutants than WT (73.4°C): 63.5°C for F26Y, 59.3°C for F26W, and 56.4°C for F26A (Table 1). The *T*_m_ values are consistent with the above temperature-induced unfolding of SpTrx WT and F26 mutants.

### Conformational flexibilities of SpTrx WT and the mutants

**Fig 6:**
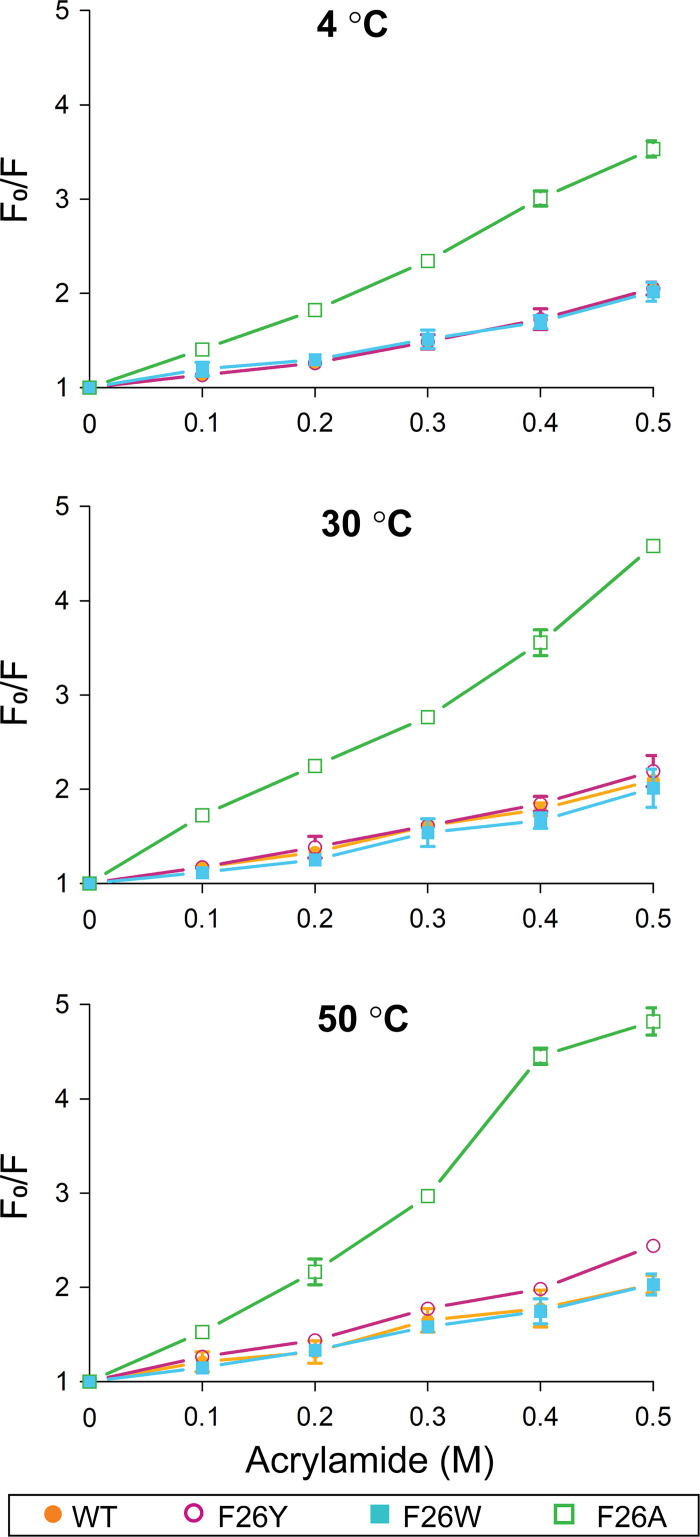
Acrylamide Stern-Volmer plot of SpTrx WT and F26 mutants. Changes in fluorescence intensities at distinct concentrations of acrylamide are presented as the ratio of the maximum fluorescence intensity without acrylamide (F_0_) to that under increasing acrylamide concentrations (0.1–0.5 M). The data presented are the means ± S.D. of three experiments.

The conformational flexibilities of SpTrx WT and F26 mutants were determined by acrylamide-induced quenching of the intrinsic protein fluorescence. The Stern-Volmer plot indicated that no changes in conformational flexibility of the overall protein structure of WT, F26Y and F26W at 4°C and 30°C (Fig 6). On the other hand, the F26Y mutant showed a higher F_0_/F value at 50°C than WT and F26W. The inverse Stern-Volmer quenching constant (*K*_D_^-1^) of F26Y decreased from 0.43 M at 30°C to 0.36 M at 50°C (Table 2). The *K*_D_^-1^ values of WT and F26W showed almost no changes at 30–50°C (Fig 6 and Table 2). Hence, the F26Y mutant had a larger conformational change than WT and F26W as it has greater relative accessibility to acrylamide at 50°C. In particular, the fluorescence intensity of F26A was largely quenched over the 4–50°C temperature range at low acrylamide concentrations, with half of the fluorescence intensity lost at 0.19 M acrylamide at 4°C (Table 2). These results suggest that a mutation of F26 to alanine with a methyl group resulted in structural instability of the β-sheet core.

**Table 2:** Inverse Stern-Volmer quenching constant, *K*_D_^-1^ (M).

	*K*_D_^-1^ (M)		
	4°C	30°C	50°C
WT	0.48 ± 0.04	0.46 ± 0.05	0.49 ± 0.05
F26Y	0.48 ± 0.04	0.43 ± 0.05	0.36 ± 0.01
F26W	0.52 ± 0.06	0.51 ± 0.1	0.49 ± 0.06
F26A	0.19 ± 0.01	0.15 ± 0.01	0.12 ± 0.01

*K*_D_^-1^ is the acrylamide concentration at which 50% of the fluorescence intensity is quenched.

### CD analysis of SpTrx WT and mutants

**Fig 7:**
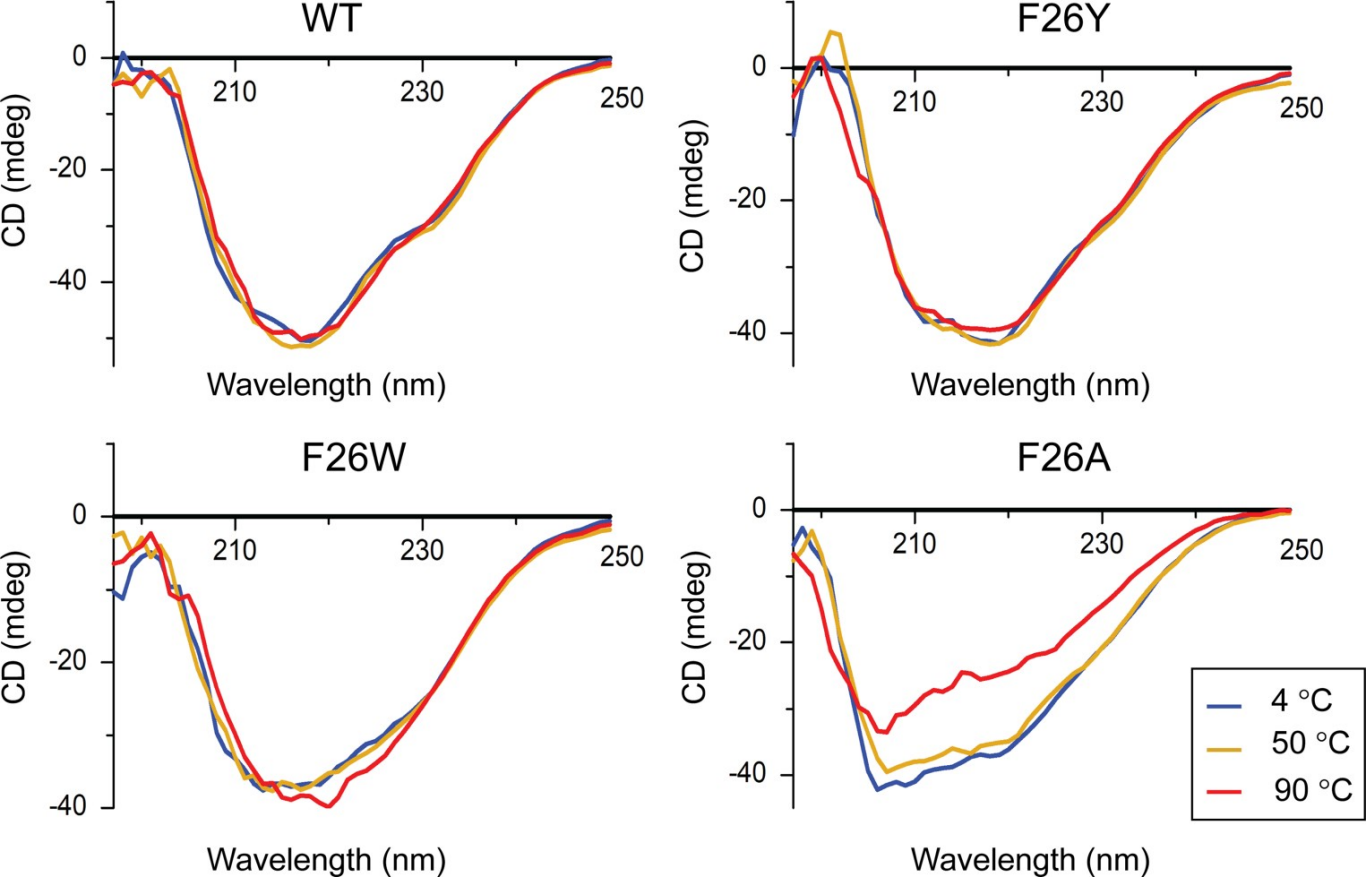
CD spectra of SpTrx WT and F26 mutants. Temperature-induced unfolding of the secondary structures of the proteins was evaluated by measuring CD spectra at 25°C following incubation of the sample (0.3 mg/mL) at 4, 50, and 90°C for 2 h.

The effects of F26 mutations on the secondary structure of SpTrx were examined by incubating the WT and F26 mutant proteins at different temperatures (4°C, 50°C, and 90°C) for 2 h before measuring the CD spectra. WT, F26Y, and F26W maintained their secondary structures in the 4–90°C temperature range with similar α-helical content (approximately 68–71%), whereas F26A exhibited increased α-helical content (80.5%) at 4°C and underwent a substantial structural change at 90°C with a 75.0% α-helical content (Fig 7 and S2 Table).

### Enzyme kinetics of SpTR for SpTrx WT and mutants

The kinetic parameters of SpTR were measured against the SpTrx WT and F26 mutants as substrates (Table 3). At the optimal temperature of SpTR (30°C), SpTR showed similar *K*_m_ values to SpTrx WT, F26Y, and F26W (0.63, 0.60, and 0.66 μM, respectively). On the other hand, the *k*_cat_ values of SpTR for F26Y and F26W were 46% and 31% lower relative to the *k*_cat_ value for WT (137.2 min^-1^), respectively, which led to lower kinetic efficiencies of SpTR for F26Y and F26W. SpTR showed a 10-fold higher *K*_m_ value to F26A compared to the other mutants. At 4°C, SpTR showed approximately 50% maximal activity (S4 Fig), which led to a lower catalytic rate than at 30°C. On the other hand, the *K*_m_ value of F26A did not change, suggesting that the misfolding of F26A led to a significant increase in *K*_*m*_ value, even at low temperatures. Overall, substitutions of tyrosine and tryptophan for F26 did not affect the binding affinity of SpTrx to SpTR, but the resulting conformational changes to the SpTrx structure affected its active conformation, which led to a decrease in the catalytic rates of SpTR for F26Y and F26W.

**Table 3:** Kinetic profiles of SpTR for SpTrx WT and F26 mutants as substrates.

	4°C			30°C		
	*K*_m_ (μM)	*k*_cat_ (min^-1^)	*k*_cat_/*K*_m_ (min^-1·^μM^-1^)	*K*_m_ (μM)	*k*_cat_ (min^-1^)	*k*_cat_/*K*_m_ (min^-1·^μM^-1^)
WT	1.10 ± 0.1	37.5 ± 2.6	34	0.63 ± 0.05	137.2 ± 4.9	218
F26Y	1.17 ± 0.4	21.1 ± 1.6	18	0.60 ± 0.05	74 ± 1.3	123
F26W	1.24 ± 0.2	30.0 ± 2.1	25	0.66 ± 0.04	95.8 ± 2.0	145
F26A	6.8 ± 0.9	34.7 ± 3.2	5	6.41 ± 0.30	134 ± 9.5	21

The data presented are the means ± S.D. of three experiments.

### Effect of F26 mutations on insulin reduction activity of SpTrx

**Fig 8:**
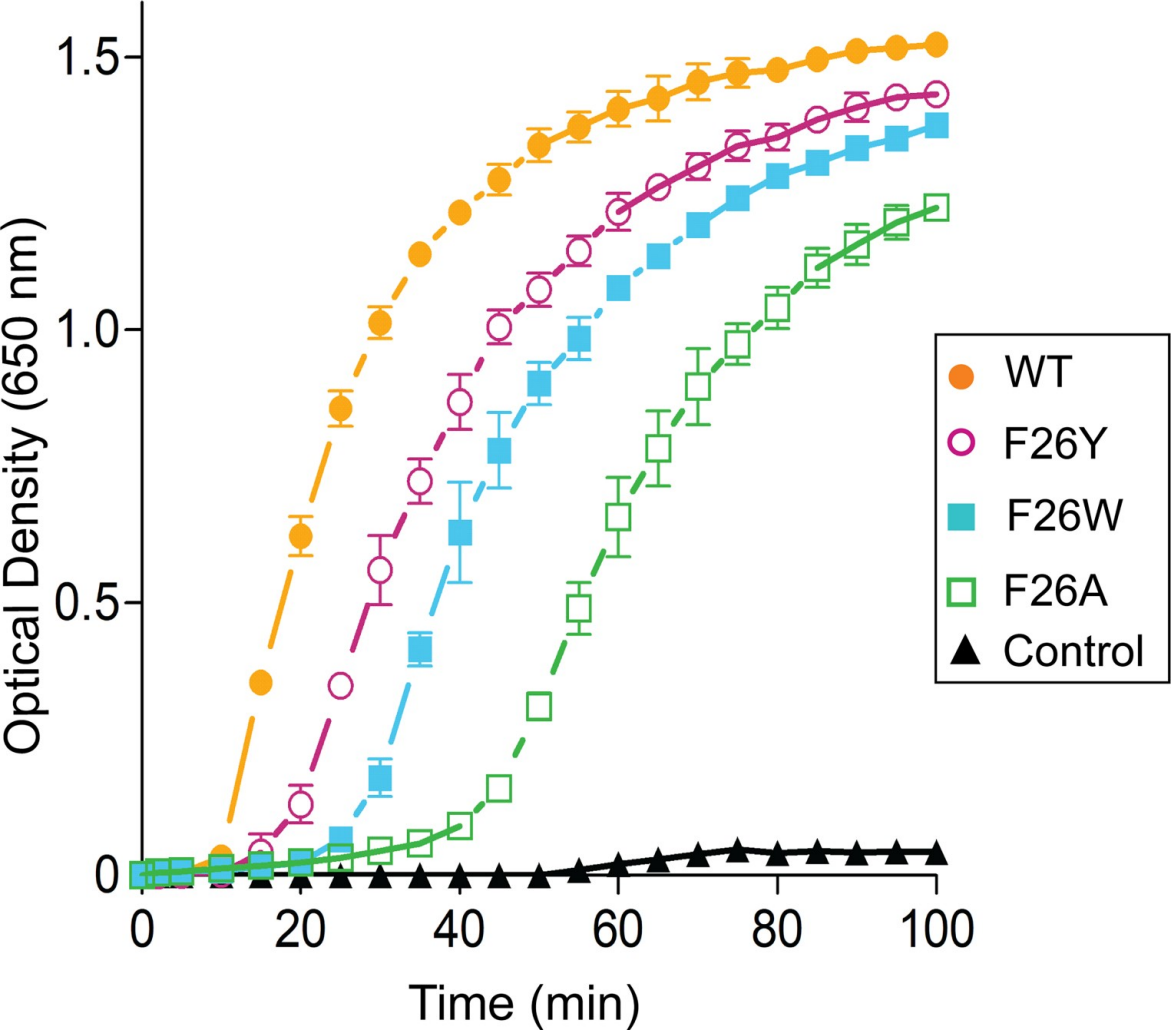
SpTrx WT and mutant-catalyzed insulin reduction by dithiothreitol. The β-chain aggregation of insulin caused by Trxs and dithiothreitol after 100 min of incubation with an appropriate amount of Trxs with 0.13 mM insulin and 0.33 mM dithiothreitol in 50 mM potassium phosphate, pH 7.2, and 2 mM EDTA. The control sample was an absence of SpTrx protein. The data presented are the means ± S.D. of three experiments.

The protein disulfide reductase activity of SpTrx WT and F26 mutants was examined by measuring insulin precipitation at 25°C at a wavelength of 650 nm. WT showed the highest insulin reduction, followed by F26Y > F26W > F26A (Fig 8). The log phase of WT began at 10 min into the precipitation reaction, whereas F26Y, F26W, and F26A entered the log phase at 20, 25, and 45 min into the precipitation reaction, respectively. Furthermore, the quantity of the β-chain insulin produced by breaking disulfide bonds in the presence of dithiothreitol reached equilibrium after a 100 min-reaction. The maximum insulin reduction activities of F26Y, F26W, and F26A were reduced by 6%, 10%, and 20% compared to WT, respectively (Fig 8). These results suggest that a mutation of F26 to tyrosine or tryptophan had a minor effect on the insulin reduction of SpTrx. Interestingly, F26A showed approximately 80% of the oxidoreductase activity of WT despite its reduced chemical and thermal stabilities.

## Discussion

Proteins adapted to cooler environments undergo structural modifications, including an increase in the glycine/proline ratio, extension of the loop length, weaker hydrophobicity, and an increase in the lysine/arginine ratio, all of which led to enhanced conformational flexibility [24–26]. As a result, cold-adapted enzymes exhibit high catalytic activity at colder temperatures but are susceptible to denaturation at moderate temperatures [27]. On the other hand, there are exceptions. Some Trxs and glutathione reductases from psychrophilic organisms exhibit high thermal stability [13, 14, 28].

The aromatic cluster of SpTrx established by four aromatic residues is close to the catalytic CXXC motif and is important for the active conformation of Trx [15]. These results showed that the substitutions of F26 to tyrosine and tryptophan in SpTrx resulted in structural instability because of the hydrophilic functional groups (tyrosine hydroxyl group or tryptophan indole N–H moiety) and led to reduced protein disulfide reduction activity and reduced catalytic rates of SpTR against F26 mutants as substrates. The expression of F26A mostly in the inclusion bodies suggests that the aromatic residue in this position is also necessary for folding SpTrx. A similar result was reported for substituting the corresponding F25 to tyrosine and alanine in mesophilic RsTrx [29], whereby the F25Y mutation caused a slightly faster urea-induced unfolding to WT, and the F25A mutation caused a 100-fold reduced protein expression, respectively.

Multiple sequence alignment (S2 Fig) showed that some Trxs possess a tyrosine residue in the corresponding position of F26, as shown in the Trx of psychrotrophic *Pseudomonas mandelii* (PmTrx) or mesophilic *Cutibacterium acnes* (CaTrx) [30, 31]. Although the buried tyrosine in the aromatic cluster of PmTrx (Y29) or CaTrx (Y27) is desirable for conformational flexibility in cooler environments, the question arises regarding how these Trxs maintain the structural stability required for their active conformations. Sequence analysis and structural modeling suggested that Y29 of PmTrx could form a hydrogen bond with a histidine residue (H72, corresponding to F69 in SpTrx) on the protein surface, thereby stabilizing the aromatic cluster (S5 Fig). These observations suggest that while the buried phenylalanine is highly preferred for the structural stability of Trxs, evolution has allowed a stability-flexibility trade-off with the presence of tyrosine, and in this case, via the formation of a hydrogen bond with another residue on the Trx surface.

Subtle adjustment of the aromatic residues in the enzyme active sites is crucial to maintaining proper conformations of cold-adapted enzymes at colder temperatures despite their intrinsically flexible structures [32–35]. For example, serine esterases use tyrosine or tryptophan in the active site wall to form a hydrogen bond with another residue in the catalytic histidine loop for active-site stabilization [32, 33, 35]. Psychrophilic esterases prefer tyrosine for conformational flexibility, whereas psychrotrophic or mesophilic esterases prefer the more hydrophobic tryptophan for structural stability [32, 33, 35]. On the other hand, glucose 6-phosphate dehydrogenase (G6PD) isozymes choose tyrosine or phenylalanine in the substrate-binding pocket depending on the metabolic pathways [34]. G6PD1, which is involved in the Entner-Doudoroff pathway, prefers tyrosine, which can form a hydrogen bond with the phosphate group of glucose 6-phosphate, for its enzymatic activity [34]. By contrast, G6PD2, which is involved in the oxidative pentose phosphate pathway, prefers purely hydrophobic phenylalanine for its thermal stability [34].

The present study showed that SpTrx and SpTR co-evolved with distinct cold adaptation patterns during evolution, and this pattern of heat-stable Trx and heat-labile TR has been observed [13, 14]. Trxs have retained their Trx-fold structure for four billion years, but psychrophilic Trx underwent structural changes during evolution to a cooler environment [10]. This is supported by the results of a reduced *T*_m_ value of 73.4°C for SpTrx compared to approximately 123°C for thermophilic Trxs [10]. The high cellular concentrations of Trxs, e.g., ~10,000 copies in *E*. *coli* [3], suggest that Trxs serve as substrates of TRs and maintain its affinity to multiple electron acceptors [36]. In contrast, TRs underwent diverse changes in their structure throughout evolution to adapt to a cooler temperature. The structural diversity of TRs is represented by different sizes, cofactors, active site motifs, and coenzymes [37].

In conclusion, this study sheds light on the roles of buried phenylalanine in the structural stability of SpTrx during adaptations to colder temperatures. Further studies on the co-evolution of TR, Trx, and downstream oxidoreductases will elucidate the cold adaptation mechanism of Trx systems in psychrophilic microorganisms.

## Supporting information

S1 Table List of primers for cloning into a TA vector and site-directed mutagenesis. (PDF)

S2 Table Alpha-helical content of SpTrx WT and mutants. (PDF)

S1 Fig Purification and molecular weight determination of SpTrx WT and mutants. (PDF)

S2 Fig Multiple sequence alignment of Trxs. (PDF)

S3 Fig Fluorescence intensities of oxidized SpTrx WT (A) and reduced SpTrx WT (B).
(PDF)

S4 Fig Optimum temperature of SpTR. (PDF)

S5 Fig Structural model of Trx from psychrotrophic *Pseudomonas mandelii* (PmTrx). (PDF)

S1 Raw images (PDF)

## References

[1] Holmgren A. Thioredoxin. Annu Rev Biochem. 1985;54:237–71. https://10.1146/annurev.bi.54.070185.001321. doi: 10.1146/annurev.bi.54.070185.001321 . 3896121

[2] Collet JF, Messens J. Structure, function, and mechanism of thioredoxin proteins. Antioxid Redox Signal. 2010;13(8):1205–16. https://10.1089/ars.2010.3114. doi: 10.1089/ars.2010.3114 . 20136512

[3] Holmgren A. Thioredoxin and glutaredoxin systems. J Biol Chem. 1989;264(24):13963–6. https://10.1016/S0021-9258(18)71625-6. . 2668278

[4] Arner ES, Holmgren A. Physiological functions of thioredoxin and thioredoxin reductase. Eur J Biochem. 2000;267(20):6102–9. https://10.1046/j.1432-1327.2000.01701.x. doi: 10.1046/j.1432-1327.2000.01701.x . 11012661

[5] Sengupta R, Holmgren A. Thioredoxin and glutaredoxin-mediated redox regulation of ribonucleotide reductase. World J Biol Chem. 2014;5(1):68–74. https://10.4331/wjbc.v5.i1.68. doi: 10.4331/wjbc.v5.i1.68 . 24600515 PMC3942543

[6] Lowther WT, Brot N, Weissbach H, Honek JF, Matthews BW. Thiol-disulfide exchange is involved in the catalytic mechanism of peptide methionine sulfoxide reductase. Proc Natl Acad Sci U S A. 2000;97(12):6463–8. https://10.1073/pnas.97.12.6463. doi: 10.1073/pnas.97.12.6463 . 10841552 PMC18625

[7] Holmgren A, Soderberg BO, Eklund H, Branden CI. Three-dimensional structure of *Escherichia coli* thioredoxin-S2 to 2.8 A resolution. Proc Natl Acad Sci U S A. 1975;72(6):2305–9. https://10.1073/pnas.72.6.2305. doi: 10.1073/pnas.72.6.2305 . 1094461 PMC432746

[8] Martin JL. Thioredoxin—a fold for all reasons. Structure. 1995;3(3):245–50. doi: 10.1016/s0969-2126(01)00154-x . 7788290

[9] Ingles-Prieto A, Ibarra-Molero B, Delgado-Delgado A, Perez-Jimenez R, Fernandez JM, Gaucher EA, et al. Conservation of protein structure over four billion years. Structure. 2013;21(9):1690–7. https://10.1016/j.str.2013.06.020. doi: 10.1016/j.str.2013.06.020 . 23932589 PMC3774310

[10] Tzul FO, Vasilchuk D, Makhatadze GI. Evidence for the principle of minimal frustration in the evolution of protein folding landscapes. Proc Natl Acad Sci U S A. 2017;114(9):E1627–E32. https://10.1073/pnas.1613892114. doi: 10.1073/pnas.1613892114 . 28196883 PMC5338549

[11] Ruggiero A, Smaldone G, Esposito L, Balasco N, Vitagliano L. Loop size optimization induces a strong thermal stabilization of the thioredoxin fold. FEBS J. 2019;286(9):1752–64. https://10.1111/febs.14767. doi: 10.1111/febs.14767 . 30675750

[12] Modi T, Huihui J, Ghosh K, Ozkan SB. Ancient thioredoxins evolved to modern-day stability-function requirement by altering native state ensemble. Philos Trans R Soc Lond B Biol Sci. 2018;373(1749). https://10.1098/rstb.2017.0184. doi: 10.1098/rstb.2017.0184 . 29735738 PMC5941179

[13] Cotugno R, Rosaria Ruocco M, Marco S, Falasca P, Evangelista G, Raimo G, et al. Differential cold-adaptation among protein components of the thioredoxin system in the psychrophilic eubacterium *Pseudoalteromonas haloplanktis* TAC 125. Mol Biosyst. 2009;5(5):519–28. https://10.1039/b818467d. doi: 10.1039/b818467d . 19381366

[14] Falasca P, Evangelista G, Cotugno R, Marco S, Masullo M, De Vendittis E, et al. Properties of the endogenous components of the thioredoxin system in the psychrophilic eubacterium *Pseudoalteromonas haloplanktis* TAC 125. Extremophiles. 2012;16(3):539–52. https://10.1007/s00792-012-0453-0. doi: 10.1007/s00792-012-0453-0 . 22527046

[15] Eklund H, Gleason FK, Holmgren A. Structural and functional relations among thioredoxins of different species. Proteins. 1991;11(1):13–28. https://10.1002/prot.340110103. doi: 10.1002/prot.340110103 . 1961698

[16] Hellinga HW, Wynn R, Richards FM. The hydrophobic core of *Escherichia coli* thioredoxin shows a high tolerance to nonconservative single amino acid substitutions. Biochemistry. 1992;31(45):11203–9. https://10.1021/bi00160a034. doi: 10.1021/bi00160a034 . 1445859

[17] Ladbury JE, Wynn R, Thomson JA, Sturtevant JM. Substitution of charged residues into the hydrophobic core of *Escherichia coli* thioredoxin results in a change in heat capacity of the native protein. Biochemistry. 1995;34(7):2148–52. https://10.1021/bi00007a007. doi: 10.1021/bi00007a007 . 7857925

[18] Lee H, Shin SC, Lee J, Kim SJ, Kim BK, Hong SG, et al. Genome sequence of *Sphingomonas* sp. strain PAMC 26621, an Arctic-lichen-associated bacterium isolated from a *Cetraria* sp. J Bacteriol. 2012;194(11):3030. https://10.1128/JB.00395-12. doi: 10.1128/JB.00395-12 . 22582384 PMC3370613

[19] Starks CM, Francois JA, MacArthur KM, Heard BZ, Kappock TJ. Atomic-resolution crystal structure of thioredoxin from the acidophilic bacterium *Acetobacter aceti*. Protein Sci. 2007;16(1):92–8. https://10.1110/ps.062519707. doi: 10.1110/ps.062519707 . 17192591 PMC2222842

[20] Madeira F, Park YM, Lee J, Buso N, Gur T, Madhusoodanan N, et al. The EMBL-EBI search and sequence analysis tools APIs in 2019. Nucleic Acids Res. 2019;47(W1):W636–W41. https://10.1093/nar/gkz268. doi: 10.1093/nar/gkz268 . 30976793 PMC6602479

[21] Slaby I, Cerna V, Jeng MF, Dyson HJ, Holmgren A. Replacement of Trp28 in *Escherichia coli* thioredoxin by site-directed mutagenesis affects thermodynamic stability but not function. J Biol Chem. 1996;271(6):3091–6. https://10.1074/jbc.271.6.3091. doi: 10.1074/jbc.271.6.3091 . 8621706

[22] Louis-Jeune C, Andrade-Navarro MA, Perez-Iratxeta C. Prediction of protein secondary structure from circular dichroism using theoretically derived spectra. Proteins. 2012;80(2):374–81. doi: 10.1002/prot.23188 . 22095872

[23] Lakowicz JR. Principles of fluorescence spectroscopy. 3rd ed. New York: Springer; 2006. xxvi, 954 p. p.

[24] Collins T, Margesin R. Psychrophilic lifestyles: mechanisms of adaptation and biotechnological tools. Appl Microbiol Biotechnol. 2019;103(7):2857–71. https://10.1007/s00253-019-09659-5. doi: 10.1007/s00253-019-09659-5 . 30729286

[25] Åqvist J, Isaksen GV, Brandsdal BO. Computation of enzyme cold adaptation. Nat Rev Chem. 2017;1(7):0051. https://10.1038/s41570-017-0051.

[26] De Maayer P, Anderson D, Cary C, Cowan DA. Some like it cold: understanding the survival strategies of psychrophiles. EMBO Rep. 2014;15(5):508–17. https://10.1002/embr.201338170. doi: 10.1002/embr.201338170 . 24671034 PMC4210084

[27] Lee C, Jang S-H, Chung H-S. Improving the stability of cold-adapted enzymes by immobilization. Catalysts. 2017;7(4):112. doi: 10.3390/catal7040112

[28] VuThi H, Jang SH, Lee C. Cloning and characterization of a thermostable glutathione reductase from a psychrophilic Arctic bacterium Sphingomonas sp. FEMS Microbiol Lett. 2019;366(18). https://10.1093/femsle/fnz218. doi: 10.1093/femsle/fnz218 . 31626298

[29] Assemat K, Alzari PM, Clement-Metral J. Conservative substitutions in the hydrophobic core of *Rhodobacter sphaeroides* thioredoxin produce distinct functional effects. Protein Sci. 1995;4(12):2510–6. https://10.1002/pro.5560041207. doi: 10.1002/pro.5560041207 . 8580841 PMC2143044

[30] Jang SH, Kim J, Hong S, Lee C. Genome sequence of cold-adapted *Pseudomonas mandelii* strain JR-1. J Bacteriol. 2012;194(12):3263. https://10.1128/JB.00517-12. doi: 10.1128/JB.00517-12 . 22628497 PMC3370865

[31] Hong S, Lee C, Jang SH. Purification and properties of an extracellular esterase from a cold-adapted *Pseudomonas mandelii*. Biotechnology letters. 2012;34(6):1051–55. https://10.1007/s10529-012-0866-y. doi: 10.1007/s10529-012-0866-y . 22315100

[32] Boyineni J, Kim J, Kang BS, Lee C, Jang S-H. Enhanced catalytic site thermal stability of cold-adapted esterase EstK by a W208Y mutation. Biochim Biophys Acta. 2014;1844(6):1076–82. doi: 10.1016/j.bbapap.2014.03.009 . 24667115

[33] Kashif A, Tran L-H, Jang S-H, Lee C. Roles of active-site aromatic residues in cold adaptation of *Sphingomonas glacialis* esterase EstSP1. ACS Omega. 2017;2(12):8760–9. https://10.1021/acsomega.7b01435. doi: 10.1021/acsomega.7b01435 . 31457406 PMC6645578

[34] Tran KN, Jang SH, Lee C. Effect of active-site aromatic residues Tyr or Phe on activity and stability of glucose 6-phosphate dehydrogenase from psychrophilic Arctic bacterium *Sphingomonas* sp. Biochim Biophys Acta Proteins Proteom. 2021;1869(1):140543. https://10.1016/j.bbapap.2020.140543. doi: 10.1016/j.bbapap.2020.140543 . 32966894

[35] Truongvan N, Chung H-S, Jang S-H, Lee C. Conserved tyrosine 182 residue in hyperthermophilic esterase EstE1 plays a critical role in stabilizing the active site. Extremophiles. 2016;20(2):187–93. https://10.1007/s00792-016-0812-3. doi: 10.1007/s00792-016-0812-3 . 26838013

[36] Napolitano S, Reber RJ, Rubini M, Glockshuber R. Functional analyses of ancestral thioredoxins provide insights into their evolutionary history. J Biol Chem. 2019;294(38):14105–18. https://10.1074/jbc.RA119.009718. doi: 10.1074/jbc.RA119.009718 . 31366732 PMC6755812

[37] Balsera M, Buchanan BB. Evolution of the thioredoxin system as a step enabling adaptation to oxidative stress. Free Radic Biol Med. 2019;140:28–35. https://10.1016/j.freeradbiomed.2019.03.003. doi: 10.1016/j.freeradbiomed.2019.03.003 . 30862542

