# Supplementary material for: Essential roles of buried phenylalanine in the structural stability of thioredoxin from a psychrophilic Arctic bacterium Sphingomonas sp
Source: PLoS One. 2021 Dec 15;16(12):e0261123. doi: 10.1371/journal.pone.0261123 (PMC8673628; doi:10.1371/journal.pone.0261123)
Supplement: S1 Table — (PDF) [file pone.0261123.s001.pdf]

Table S1. List of primers for cloning into a TA vector and site-directed mutagenesis.

		Primer sequence
SpTrx WT	Forward	5′-ccatggcgaccaagaagatcaccgacg-3′ (*Nco* I site underlined)
	Reverse	5′-aagcttcgctagctcgccctcga-3′ (*Hind* III site underlined)
F26Y	Forward	5′-ctcgtcgacTATtgggc-3′
	Reverse	5′-gcccaATAgtcgacgag-3′
F26W	Forward	5′-tcgacTGGtgggcggaat-3′
	Reverse	5′-ccaCCAgtcgacgagaac-3′
F26A	Forward	5′-ttctcgtcgacGCTtgg-3′
	Reverse	5′-attccgcccaAGCgtcg-3′
F69Y	Forward	5′-caagTATggcgtccgtgg-3′
	Reverse	5′-gacgccATActtgcccg-3′
F80Y	Forward	5′-cgatgattttgTATaagaa-3′
	Reverse	5′-cccgttcttATAcaaaat-3′
SpTR WT	Forward	5′-ccatggtgacccataccacccgcatg-3′ (*Nco* I site underlined)
	Reverse	5′-ctcgagctccgccgcctcggcca-3′ (*Xho* I site underlined)

Mutated nucleotides are shown in capital letters.
