# Supplementary material for: Essential roles of buried phenylalanine in the structural stability of thioredoxin from a psychrophilic Arctic bacterium Sphingomonas sp
Source: PLoS One. 2021 Dec 15;16(12):e0261123. doi: 10.1371/journal.pone.0261123 (PMC8673628; doi:10.1371/journal.pone.0261123)
Supplement: S2 Table — (PDF) [file pone.0261123.s002.pdf]

Table S2. Alpha-helical content of SpTrx WT and mutants.

	Alpha-helical content (%)			
Temperature (°C)	WT	F26Y	F26W	F26A
4	71.8	69.3	68.8	80.5
50	71.3	68.9	67.2	78.2
90	71.3	70.7	68.2	75.0

The α-helical content was analyzed from the CD spectra shown in Fig. 7 using the K2D3 server.
