# Supplementary material for: Essential roles of buried phenylalanine in the structural stability of thioredoxin from a psychrophilic Arctic bacterium Sphingomonas sp
Source: PLoS One. 2021 Dec 15;16(12):e0261123. doi: 10.1371/journal.pone.0261123 (PMC8673628; doi:10.1371/journal.pone.0261123)
Supplement: S1 Fig — (PDF) [file pone.0261123.s003.pdf]

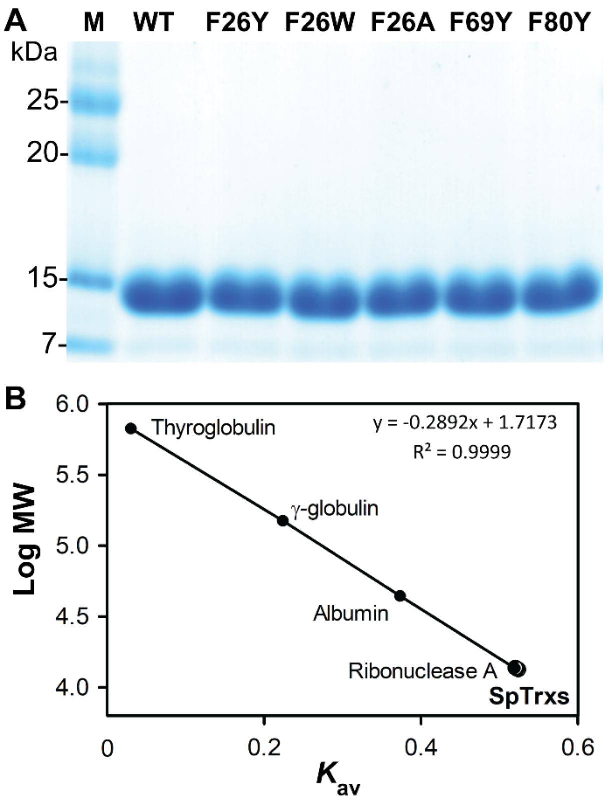

S1 Fig. Purification and molecular weight determination of SpTrx WT and mutants. (A) Sodium dodecyl sulfate-polyacrylamide gel electrophoresis of WT and mutants. M, Marker. (B) Determination of the molecular weight of native SpTrx and mutants by size-exclusion chromatography on a Superdex 200 10/300 GL column. Bovine thyroglobulin (670 kDa), γ-globulin (150 kDa), albumin (44.3 kDa), and ribonuclease A (13.7 kDa).
