# Supplementary material for: Essential roles of buried phenylalanine in the structural stability of thioredoxin from a psychrophilic Arctic bacterium Sphingomonas sp
Source: PLoS One. 2021 Dec 15;16(12):e0261123. doi: 10.1371/journal.pone.0261123 (PMC8673628; doi:10.1371/journal.pone.0261123)
Supplement: S2 Fig — (PDF) [file pone.0261123.s004.pdf]

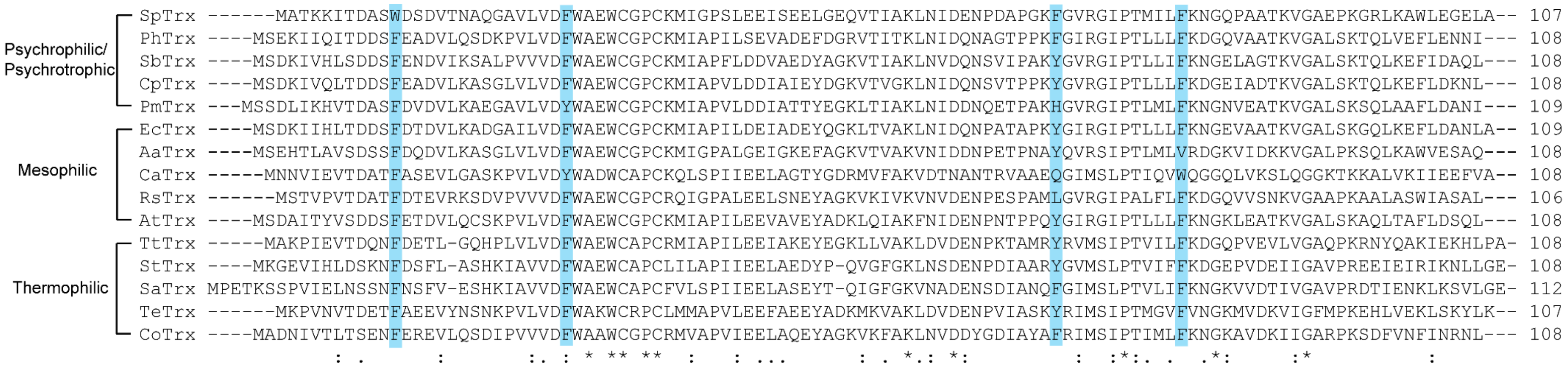

S2 Fig. Multiple sequence alignment of Trxs. Psychrophilic Trxs: SpTrx (*Sphingomonas* sp. PAMC 26621), PhTrx (*Pseudoalteromonas haloplanktis*), SbTrx (*Shewanella benthica*), and CpTrx (*Colwellia piezophila*). Psychrotrophic Trx: PmTrx (*Pseudomonas mandelii*). Mesophilic Trxs: EcTrx (*Escherichia coli*), AaTrx (*Acetobacter aceti*), CaTrx (*Cutibacterium acnes*), RsTrx (*Rhodobacter sphaeroides*), and AtTrx (*Acidithiobacillus thiooxidans*). Thermophilic Trxs: TtTrx (*Thermus thermophilus*), StTrx (*Sulfurisphaera tokodaii*), SaTrx (*Sulfolibus acidocaldarius*), TeTrx (*Thermoanaerobacter ethanolicus*), and CoTrx (*Caldicellulosiruptor obsidiansis*).
