# Supplementary figures and images for: Essential roles of buried phenylalanine in the structural stability of thioredoxin from a psychrophilic Arctic bacterium Sphingomonas sp
Source: PLoS One. 2021 Dec 15;16(12):e0261123. doi: 10.1371/journal.pone.0261123 (PMC8673628; doi:10.1371/journal.pone.0261123)

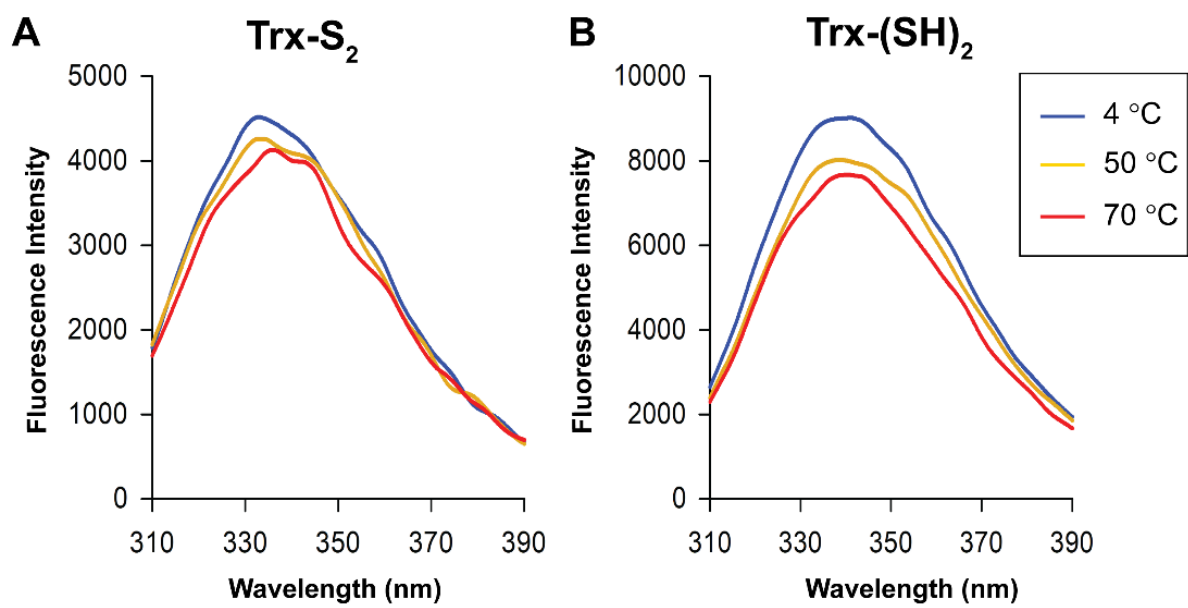

**S3 Fig. Fluorescence intensities of oxidized SpTrx WT (A) and reduced SpTrx WT (B).**

Supplement: S3 Fig — Fluorescence intensities of oxidized SpTrx WT (A) and reduced SpTrx WT (B). (PDF) [file pone.0261123.s005.pdf]

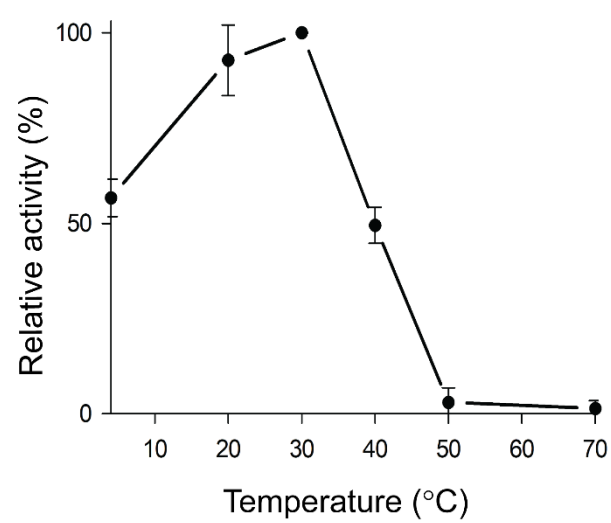

**S4 Fig. Optimum temperature of SpTR.**

Supplement: S4 Fig — (PDF) [file pone.0261123.s006.pdf]
