# Supplementary material for: Essential roles of buried phenylalanine in the structural stability of thioredoxin from a psychrophilic Arctic bacterium Sphingomonas sp
Source: PLoS One. 2021 Dec 15;16(12):e0261123. doi: 10.1371/journal.pone.0261123 (PMC8673628; doi:10.1371/journal.pone.0261123)
Supplement: S5 Fig — (PDF) [file pone.0261123.s007.pdf]

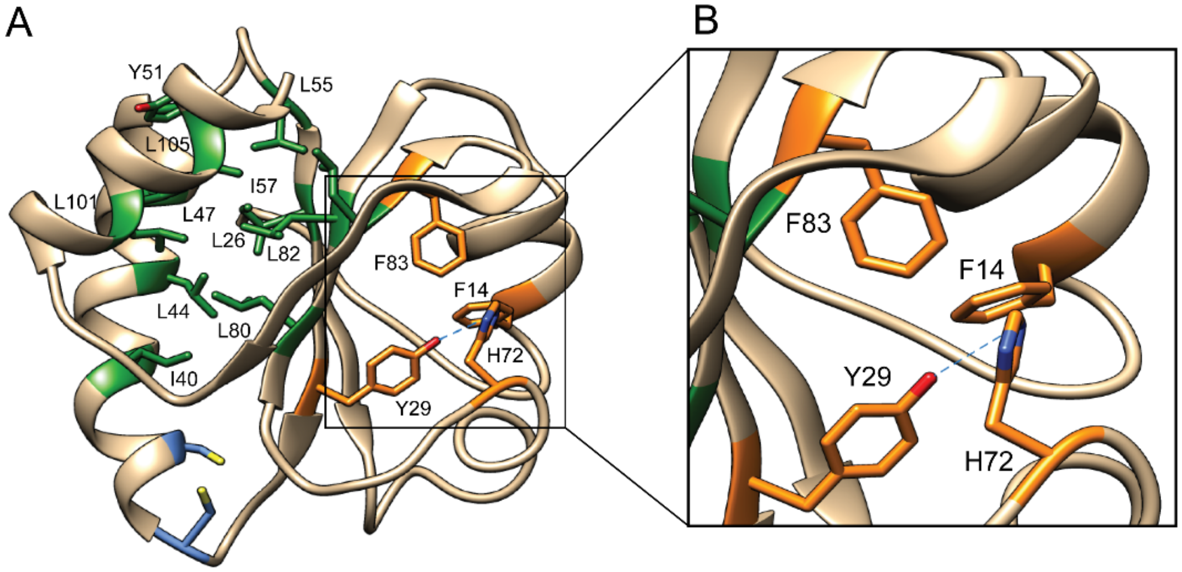

S5 Fig. Structural model of Trx from psychrotrophic *Pseudomonas mandelii* (PmTrx). (A) The ribbon diagram of PmTrx. The structural model of PmTrx (NCBI ID: OYQ15722.1) was constructed at the Swiss-Model based on the crystal structure of *E. coli* Trx (PDB ID: 2H72). The hydrophobic core of PmTrx consisted of the aliphatic cluster (green) and the aromatic cluster (orange). Two catalytic cysteine residues are shown in blue. (B) Enlarged view of the PmTrx aromatic cluster. The hydroxyl group of Y29 forms a hydrogen bond with the imidazole group of H72 located on the surface of PmTrx.
