# Supplementary material for: Essential roles of buried phenylalanine in the structural stability of thioredoxin from a psychrophilic Arctic bacterium Sphingomonas sp
Source: PLoS One. 2021 Dec 15;16(12):e0261123. doi: 10.1371/journal.pone.0261123 (PMC8673628; doi:10.1371/journal.pone.0261123)
Supplement: S1 Raw images — (PDF) [file pone.0261123.s008.pdf]

**S1 Raw images.**

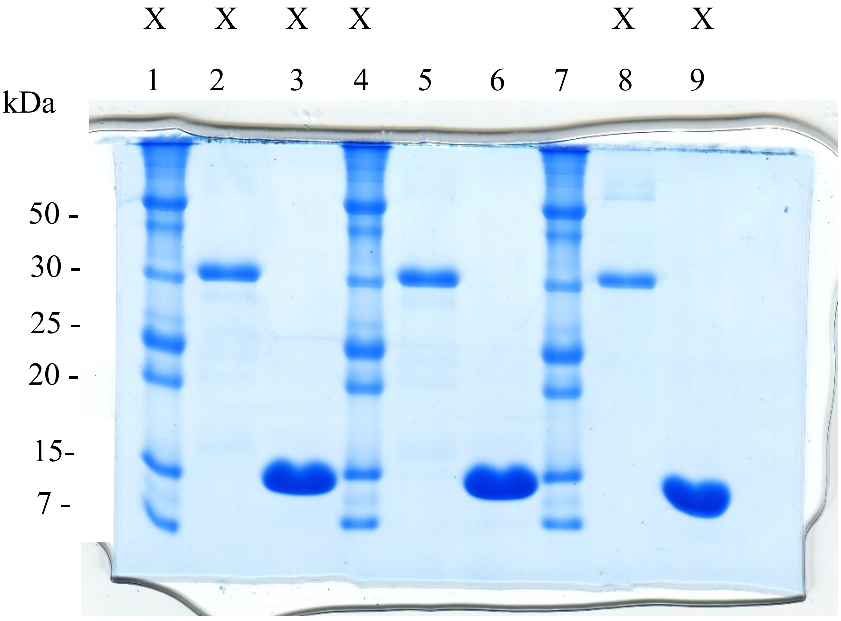


**Fig 2A.** SDS-polyacrylamide gel image of WT SpTrx and SpTR.

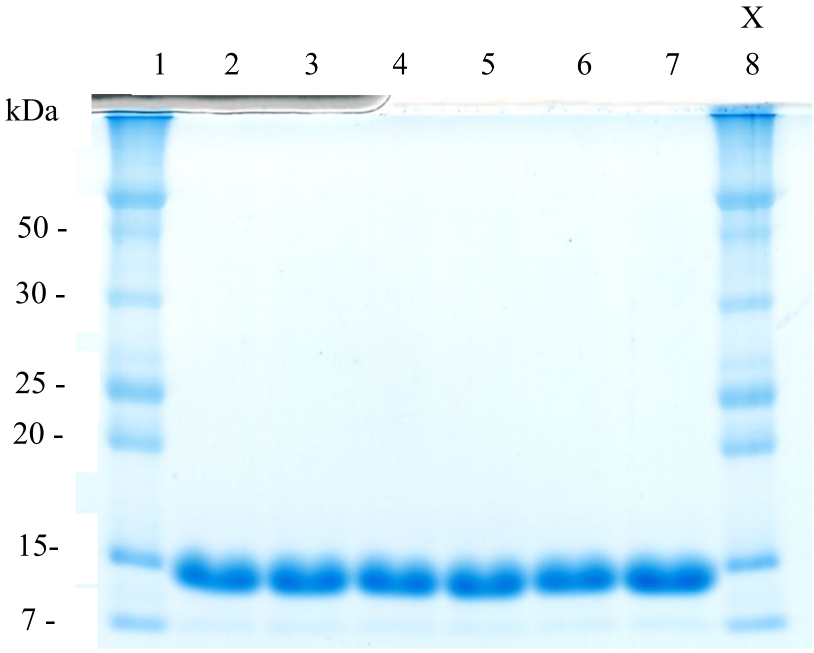


**S1 Fig.** SDS-polyacrylamide gel image of SpTrx WT and mutants.
